# The effects of levosimendan in patients undergoing transcatheter aortic valve replacement- a retrospective analysis

**DOI:** 10.3389/fphar.2022.969088

**Published:** 2022-11-03

**Authors:** Zhenyan Zhao, Zhen Meng, Guangyuan Song, Chunrong Wang, Sheng Shi, Jie Zhao, Hongliang Zhang, Moyang Wang, Guannan Niu, Zheng Zhou, Jianhui Wang, Yongjian Wu

**Affiliations:** ^1^ State Key Laboratory of Cardiovascular Disease, Department of Cardiology, Fuwai Hospital, National Center for Cardiovascular Disease, Chinese Academy of Medical Science and Peking Union Medical College, Beijing, China; ^2^ Department of Cardiology, China-Japan Friendship Hospital, Beijing, China; ^3^ Interventional Center of Valvular Heart Disease, Beijing Anzhen Hospital, Capital Medical University, Beijing Institute of Heart, Lung and Blood Vessel Diseases, Beijing, China; ^4^ Department of Anesthesiology, Fuwai Hospital, Chinese Academy of Medical Sciences and Peking Union Medical College, Beijing, China

**Keywords:** levosimendan, aortic stenosis, transcatheter valve replacement, inotrope, heart failure

## Abstract

**Background:** Aortic stenosis (AS) increases left ventricular afterload, leading to cardiac damage and heart failure (HF). Transcatheter aortic valve replacement (TAVR) is an effective therapy for AS. No inotropic agents including levosimendan have been evaluated in patients undergoing TAVR.

**Methods:** A total of 285 patients underwent TAVR between 2014 and 2019; 210 were included in the matched analysis and 105 received 0.1 μg/kg body weight/min levosimendan immediately after the prosthesis had been successfully implanted. Medical history, laboratory tests, and echocardiography results were analyzed. Endpoints including 2-year all-cause mortality, stroke, or HF-related hospitalization, and a combination of the above were analyzed by Cox proportional hazard models.

**Results:** The levosimendan group had no difference in 2-year mortality compared with the control group (hazard ratio [HR]: 0.603, 95% confidence interval [CI]: 0.197–1.844; *p* = 0.375). However, levosimendan reduced stroke or HF-related hospitalization (HR: 0.346; 95% CI: 0.135–0.884; *p* = 0.027) and the combined endpoint (HR: 0.459, 95% CI: 0.215–0.980; *p* = 0.044). After adjusting for multiple variants, levosimendan still reduced stroke or HF-related hospitalization (HR: 0.346, 95% CI: 0.134–0.944; *p* = 0.038).

**Conclusion:** Prophylactic levosimendan administration immediately after valve implantation in patients undergoing TAVR can reduce stroke or HF-related hospitalization but does not lower all-cause mortality.

## Introduction

Aortic stenosis (AS) is the most prevalent heart valve disorder in developed countries (Lindman et al., 2016), which is age-related with a prevalence of 0.4% in the general population and 1.7% in individuals over 65 years old (Nkomo et al., 2006). Progressive calcification of the aortic valve (AV) obstructs the left ventricular outflow tract (LVOT), leading to increased afterload of the left ventricle (LV). AS results in cardiac damage, inadequate cardiac output, decreased exercise capacity, heart failure (HF), and ultimately death if untreated (Lindman et al., 2013). To date, no drug can improve the long-term outcome of AS compared with its natural progression, and AV replacement (AVR) is the first-line therapy for patients (Boskovski and Gleason, 2021; Vahanian et al., 2021). In the past several decades, transcatheter AV replacement (TAVR) has revolutionized the treatment of AS. Recent guidelines suggest that TAVR can be considered in AS patients according to individual clinical, anatomical, and procedural characteristics, regardless of surgical risk (Vahanian et al., 2021). Even though the consensus among cardiologists is that the symptoms of AS are mainly driven by dysfunction of the LV and potential cardiac damage, no study has focused on inotropic agent use in TAVR patients during the perioperative period.

Levosimendan (Simdax, Orion) is an inotropic agent used in patients with chronic HF, cardiogenic shock, takotsubo cardiomyopathy, and cardiac surgery (Papp et al., 2020). Levosimendan enhances cardiac contractility without increasing myocardial oxygen consumption. The pharmacological mechanism of levosimendan is to increase the calcium sensitivity of myocardial contractile units through binding to troponin C (Haikala et al., 1995). Additionally, levosimendan can promote vasodilation through the opening of adenosine triphosphate-dependent potassium (K_ATP_) channels on vascular smooth muscle cells (Yokoshiki et al., 1997). It can also open K_ATP_ channels on the mitochondrial inner membrane leading to cardioprotection (Kopustinskiene et al., 2004). The effects of levosimendan and its metabolite OR-1896 reportedly last up to 7 days (Erdei et al., 2006).

A recent prospective study of 185 patients who underwent mitral valve and AV replacement showed that levosimendan administration can effectively improve heart function, decrease the need for vasoactive drugs, shorten the length of intensive care unit (ICU) stay, reduce the incidence of postoperative adverse events, and promote the recovery of patients after surgery (Sheng et al., 2021). However, levosimendan administration in patients undergoing AV replacement, especially TAVR, has not been evaluated.

This is the first study to describe the effect of prophylactic levosimendan administration immediately after prosthesis implantation on the long-term survival of patients undergoing TAVR.

## Materials and methods

### Study design

This was a retrospective single-center study that described the effect of prophylactic levosimendan administration on patients undergoing TAVR. The protocol was approved by the ethical committee of Fuwai Hospital (Beijing, China), and the study was performed in accordance with the Declaration of Helsinki. AS patients undergoing TAVR between 2014 and 2019 were included, as levosimendan has not been available in our facility since 2019 due to policy reform of the essential drug directory. Emergency TAVR was excluded. All eligible patients provided written informed consent. To adjust for relevant differences in baseline characteristics, 1:1 propensity score matching (PSM) of the levosimendan group (LS+) and control group (LS-) was conducted.

### Clinical data collection

Medical records including medical history, laboratory tests, and echocardiography results were obtained from the hospital’s electronic medical records system. All laboratory tests were performed within 24 h before surgery and 48 h after operation. Echocardiography parameters were collected according to the last examination before surgery.

### Levosimendan administration

Patients received an intravenous infusion of levosimendan at a dose of 0.1 μg/kg body weight per min for 24 h immediately after the prosthesis was successfully implanted. The use of concomitant medication including other inotropes, vasopressors, mechanical circulatory support, and hemodynamic monitoring were left to the discretion of the treating physicians according to relevant guidelines.

### Echocardiography

Two-dimensional, color, pulsed- and continuous-wave Doppler images were obtained from apical and parasternal views according to current recommendations with the patients at rest in the left lateral decubitus position using commercially available ultrasound systems. Left ventricular systolic function was assessed using LV ejection fraction (LVEF) measured by the biplane modified Simpson method. From the apical 3- or 5-chamber views, continuous-wave Doppler recordings were obtained to estimate peak aortic jet velocity. Echocardiography was performed by an experienced sonologist at Fuwai Hospital.

### Laboratory testing

N-terminal pro-B-type natriuretic peptide (NT-proBNP) and cardiac troponin I (cTnI) levels were measured by the Center of Laboratory Medicine, Fuwai Hospital. Briefly, plasma NT-proBNP concentrations were determined by Roche NT-proBNP Elecsys (Roche Diagnostics, Basel, Switzerland). Serum cTnI was measured using a high-sensitivity assay (ARCHITECH STAT; Abbott Laboratories, Chicago, IL, United States) with a reference range from 0 to 0.016 ng/ml.

### Endpoints and follow-up

Follow-up was conducted annually by telephone interviewers using standardized questionnaires. Since the last patients in this study received TAVR in October 2019, we selected 2-year all-cause mortality and 2-year stroke or HF-related hospitalization as the primary endpoints. The secondary endpoint was a composite of the two primary endpoints.

### Statistical analyses

Normal distributed continuous variables are expressed as the mean ± standard deviation (SD), non-normal distributed continuous variables are expressed as the median (interquartile range, IQR), and categorical variables are expressed as counts (percentage). Differences among groups were compared using the Student’s *t*-test or Mann-Whitney U test for continuous variables according to the distribution, and the chi-square test or Fisher’s exact test for categorical variables. To adjust for potentially confounding baseline parameters between the LS+ and LS- groups, PSM was performed. Covariates included in the matching were NT-proBNP and Society of Thoracic Surgeons (STS) score. Subsequently, 1:1 nearest neighbor matching was performed. The maximum caliper between matched participants was set at 0.2. NT-proBNP and cTnI levels before and after surgery were compared between groups using repeated-measures analysis of variance (ANOVA). Long-term survival functions were determined using the Kaplan-Meier method and compared using the log-rank test. To evaluate the association of levosimendan administration and other parameters with primary and secondary endpoints, survival data were fitted using Cox proportional hazard models. Since the incidence of events was relatively small (13 patients died and 22 patients had stroke or HF-related hospitalization), we performed univariable Cox regression for variable selection, and variables with *p* < 0.10 were reserved as candidate variables for each endpoint. Age, STS score, levosimendan, and candidate variables were selected and introduced as covariates in the multivariable Cox proportional hazards models. The proportional hazards assumption was examined by inspection of Schoenfeld residuals. For both univariable and multivariable analyses, hazard ratios (HRs) with 95% confidence intervals (CIs) were presented. NT-proBNP and cTnI concentrations were log-transformed to improve normality in PSM, repeated measures ANOVA, and Cox proportional hazard models. Statistical analyses were performed using SPSS version 26.0 (IBM, Armonk, NY, United States), GraphPad Prism version 9 (GraphPad Software, Inc., La Jolla, CA, United States), and R version 4.1.0 (R Foundation for Statistical Computing, Vienna, Austria). Two-sided *p* < 0.05 was considered statistically significant.

## Results

### Clinical features and characteristics

A total of 285 patients underwent TAVR at our institution between January 2014 and October 2019. Then, 157 patients received levosimendan prophylactically and 128 patients did not receive levosimendan. Baseline for the 285 patients was presented in Supplementary Table S1. In the matched study population (n = 105 for each group), patients’ baseline characteristics were balanced between the groups except for dyslipidemia (Table 1). The distribution of cardiovascular risk factors, heart function parameters, and AS severity was similar between groups except for dyslipidemia. However, due to the wide administration of statin, patients’ low-density lipoprotein cholesterol levels between groups were similar (LS- vs. LS+: 2.48 ± 0.84 vs. 2.48 ± 0.89 mmol/L; *p* = 0.972).

**TABLE 1:** Baseline characteristics for matched study population.

Parameter	LS-N = 105	LS + N = 105	*p* Value
Age, mean (SD), years	75.70 (10.28)	76.09 (6.18)	0.739
Male sex, n (%)	64 (61.0)	66 (62.9)	0.776
BMI, mean (SD), Kg/m^2^	23.57 (3.47)	23.34 (3.50)	0.644
STS risk score, median [IQR]	4.18 [3.59]	4.40 [5.07]	0.985
Heart failure, n (%)	90 (85.7)	81 (77.1)	0.155
NYHA class, n (%)			
I, n (%)	0 (0.0)	1 (1.0)	0.127
II, n (%)	8 (7.6%)	15 (14.3)	
III, n (%)	66 (62.9)	52 (49.5)	
IV, n (%)	16 (15.2)	13 (12.4)	
Peak AV velocity, mean (SD), cm/s	4.53 (0.88)	4.52 (0.71)	0.928
LVEF, mean (SD), %	56.62 (14.78)	55.84 (14.13)	0.698
cTnI, median [IQR], ng/ml	0.025 [0.047]	0.024 [0.031]	0.088
NT-proBNP, median [IQR], pg/ml	1760.7 [3197.5]	1720.0 [3978.0]	0.884
diabetes, n (%)	29 (27.6)	34 (32.4)	0.451
CKD, n (%)	8 (7.6)	8 (7.6)	1.000
COPD, n (%)	19 (18.1)	11 (10.5)	0.115
hypertension, n (%)	60 (57.1)	61 (58.1)	0.889
dyslipidemia, n (%)	52 (49.5)	64 (61.0)	0.096
Previous CAD, n (%)	47 (44.8)	37 (35.2)	0.159
Previous AF, n (%)	21 (20)	16 (15.2)	0.365
statin, n (%)	65 (61.9)	74 (70.5)	0.189
aspirin, n (%)	70 (66.7)	53 (50.5)	0.017
β-block, n (%)	65 (61.9)	61 (58.1)	0.673
ACEI/ARB, n (%)	19 (18.1)	21 (20.0)	0.725
CCB, n (%)	13 (12.4)	17 (16.2)	0.430
digoxin, n (%)	2 (1.9)	2 (1.9)	1.000

Abbreviations: BMI, body mass index; STS, society of thoracic surgeons; NYHA, new york heart association; AV, aortic valve; LVEF, left ventricular ejection fraction; cTnI, cardiac troponin I; NT-proBNP, N-terminal pro-B-type natriuretic peptide; CKD, chronic kidney disease; COPD, chronic obstructive pulmonary disease; CAD, coronary heart disease; AF, atrial fibrillation; ACEI/ARB, angiotensin converting enzyme inhibitor/angiotensin receptor blocker; CCB, calcium channel blocker.LS-, control group; LS+, levosimendan group.

### Procedure

Patients underwent TAVR using two kinds of balloon-expanding valve including the Venus A-valve (Venus Medtech, Hangzhou, China) or TaurusOne Valve (Peijia Medical Ltd., Suzhou, China). Two patients died during surgery and four patients were converted to open surgery in the LS + group. One patient died during surgery and four patients were converted to open surgery in the LS- group. All the three patients died for circulation collapse after balloon dilatation. Using Valve Academic Research Consortium criteria, TAVR implantation was successful in 99 patients in the LS + group and 100 patients in the LS- group. There were no differences in operative mortality, conversion to open surgery, and device success among groups (*p* = 0.842). Subsequent analyses were focused on device success in patients.

### Laboratory tests and in-hospital adverse events

**FIGURE 1:**
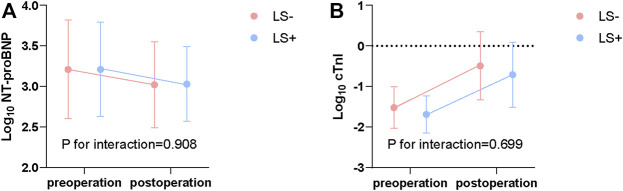
Log transformed NT-proBNP (pg/ml) **(A)** and cTnI (ng/ml) **(B)** before and after surgery. P for interaction from repeated measures ANOVA. NT-proBNP, N-terminal pro B type natriuretic peptide; cTnI, cardiac troponin I; ANOVA, Analysis of variance; LS+, Levosimendan group; LS-, Control group.

Pre- and post-operative NT-proBNP and cTnI levels are shown in Figure 1. NT-proBNP decreased after operation (LS-: 1760 [3197.5] to 1094 [1810] pg/ml, LS+: 1720 [3978.0] to1422 [1879] pg/ml; *p* < 0.001). cTnI slightly increased after operation (LS-: 0.025 [0.047] to 0.469 [1.039], LS+: 0.024 [0.031] to 0.301 [0.804]; *p* < 0.001). However, there was no evidence showing that levosimendan administration can influence the impact of surgery (*p* = 0.908 for interaction of NT-proBNP; *p* = 0.699 for interaction of cTnI).

In-hospital outcomes and echocardiographic parameters are shown in Table 2. There was no difference in peak AV velocity or LVEF between groups. Post-operative ICU stay and hospital admission were similar between groups. The relatively rare occurrences of in-hospital events weaken the power of tests evaluating the impact of levosimendan. The LS + group tended to have less acute kidney injury (LS- vs. LS+: 6 vs. 3; *p* = 0.299) and major adverse cardiovascular events (death, stroke, cardiac arrest, acute HF) (LS- vs. LS+: 10 vs. 3; *p* = 0.043). It is worth noting that one patient in the LS + group underwent hypotension, which is considered a main side effect of levosimendan. However, the frequency (<1.0%) seemed tolerable. Ten patients in each group received permanent pacemaker implantation, with no difference between the LS+ and LS- groups. No patients suffered from a major bleeding event. Several patients had minor bleeding at the puncture site or melena, but the medical record on these events was insufficient.

**TABLE 2:** In-hospital outcomes and echocardiographic parameters.

Parameter	LS-N = 99	LS + N = 100	*p* Value
Length of stay, median [IQR], day	7.0 [6]	8.0 [6]	0.107
ICU, n (%)	17 (17.2)	18 (18.0)	0.878
death, n (%)	2 (2.0)	0 (0.0)	0.153
stroke, n (%)	2 (2.0)	0 (0.0)	0.153
Cardiac arrest, n (%)	3 (3.0)	1 (1.0)	0.308
AHF, n (%)	3 (3.0)	2 (2.0)	0.642
MACE, n (%)	10 (10.1)	3 (3.0)	0.043
hypotension, n (%)	1 (1.0)	0 (0.0)	0.314
AKI, n (%)	6 (6.1)	3 (3.0)	0.299
Peak AV velocity, mean (SD), cm/s	2.25 (0.54)	2.38 (0.53)	0.109
LVEF, mean (SD), %	57.83 (9.99)	57.98 (12.28)	0.927

Abbreviations: ICU, intensive care unit; AHF, acute heart failure; MACE, major adverse cardiovascular events (death + stroke + cardiac arrest + AHF); AKI, acute kidney injure; AV, aortic valve; LVEF, left ventricular ejection fraction. LS-, control group; LS+, levosimendan group.

### Long-term outcome and survival analysis

**FIGURE 2:**
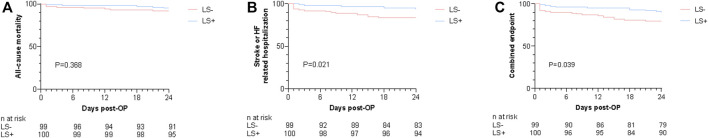
Kaplan-Meier estimates for the cumulative events rates of 2-year all-cause mortality **(A)**, 2-year stroke or HF-related hospitalization **(B)**, and combined endpoint **(C)**. *p* value from log-rank chi-square test. OP, operation; HF, Heart failure; Levosimendan group; LS-, Control group.

A 2-year follow-up was conducted for all patients. Kaplan-Meier estimation of each endpoint was applied for patients in each group as shown in Figure 2. There were no differences in long-term survival between groups (*p* = 0.368). Regarding stroke or HF-related hospitalization and the combined endpoint, the LS- group had significantly higher cumulative event rates compared with the LS + group (*p* < 0.05 for each endpoint).

The correlates of each endpoint on univariable Cox regression are shown in Table 3 and Supplementary Tables S2–4. On univariate Cox analysis, chronic obstructive pulmonary disease was associated with 2-year all cause-mortality (HR: 4.019, 95% CI: 1.315–12.289; *p* = 0.015). Diabetes (HR: 2.880, 95% CI: 1.244–6.668; *p* = 0.014), stroke (HR: 2.455, 95% CI: 0.960–6.275; *p* = 0.061) previous coronary artery disease (CAD) (HR: 2.586, 95% CI: 1.085–6.166; *p* = 0.032), previous arterial fibrillation (AF) (HR: 2.799, 95% CI: 1.174–6.676; *p* = 0.020) and levosimendan (HR: 0.346, 95% CI: 0.135–0.884; *p* = 0.027) were associated with stroke or HF-related hospitalization. Diabetes (HR: 2.392, 95% CI: 1.169–4.895; *p* = 0.017), stroke (HR: 2.462, 95% CI: 1.096–5.534; *p* = 0.029), and levosimendan (HR: 0.459, 95% CI: 0.215–0.980; *p* = 0.044) were associated with combined endpoints. The variables mentioned above and age, STS score, and levosimendan were included in the multivariable Cox regression models. After adjusting for several risk factors, levosimendan could reduce 65.4% 2-year stroke or HF-related hospitalization (95% CI: 0.134–0.944; *p* = 0.038). The LS + group tended to have a lower 2-year combined outcomes rate (HR: 0.483, 95% CI:0.221–1.056; *p* = 0.068). Levosimendan had no impact on 2-year all-cause mortality (HR: 0.736, 95% CI: 0.235–2.301; *p* = 0.598).

**TABLE 3:** Multivariable cox proportional hazard analyses.

	Hazard ratio (95% CI)	*p* Value
All-cause mortality		
Age, per 1 year increase	1.059 (0.967–1.161)	0.217
STS risk score, log	0.971 (0.779–1.209)	0.791
COPD, yes/no	3.803 (1.207–11.984)	0.023
Levosimendan, yes/no	0.736 (0.235–2.301)	0.598
Stroke or HF relative-hospitalization		
Age, per 1 year increase	1.034 (0.960–1.114)	0.376
STS risk score, log	0.943 (0.787–1.130)	0.524
CAD, yes/no	1.880 (0.769–4.595)	0.166
Diabetes, yes/no	3.426 (1.424–8.246)	0.006
Stroke, yes/no	1.785 (0.667–4.782)	0.249
Previous AF, yes/no	3.075 (1.212–7.806)	0.018
Levosimendan, yes/no	0.356 (0.134–0.944)	0.038
Combined endpoint		
Age, per 1 year increase	1.042 (0.980–1.108)	0.190
STS risk score, log	0.945 (0.809–1.105)	0.481
CAD, yes/no	1.440 (0.687–3.020)	0.334
Diabetes, yes/no	2.374 (1.143–4.931)	0.020
Stroke, yes/no	1.851 (0.799–4.289)	0.151
Levosimendan, yes/no	0.483 (0.221–1.056)	0.068

Abbreviations: STS, society of thoracic surgeons; COPD, chronic obstructive pulmonary disease; CAD, coronary heart disease; AF, atrial fibrillation; HF, heart failure.

### Subgroup analyses

Finally, we conducted different subgroup analyses for each endpoint depending on sex, LVEF ≤35%, and the existence of HF. Subgroup analyses were explorative and a forest plot could not be depicted, because sample sizes of certain subgroups were quite small and CIs were wide. The results of subgroup analyses are shown in Table 4. Briefly, female patients with HF and preserved EF (>35%) trended toward better outcomes with levosimendan; however, there was no interaction in every subgroup analysis.

**TABLE 4:** Subgroup analysis.

	n	HR (95% CI)	P	P for interaction
2-year all-cause mortality				
Heart failure				0.472
Yes	161	0.458 (0.119–1.773)	0.258	
No	38	1.264 (0.115–13.948)	0.848	
LVEF≤35%				0.968
Yes	25	52.454 (0.000-600986,101)	0.633	
No	174	0.492 (0.148–1.634)	0.247	
Sex				0.067
male	122	0.696 (0.156–3.111)	0.635	
female	77	0.513 (0.094–2.801)	0.441	
2-year stroke or heart failure hospitalization				
Heart failure				0.581
Yes	161	0.258 (0.073–0.913)	0.036	
No	38	0.433 (0.097–1.936)	0.273	
LVEF≤35%				0.960
Yes	25	52.454 (0.000-600986,101)	0.633	
No	174	0.293 (0.107–0.799)	0.017	
Sex				0.209
male	122	0.555 (0.181–1.696)	0.301	
female	77	0.123 (0.015–0.988)	0.049	
2-year Combined endpoint				
Heart failure				0.608
Yes	161	0.384 (0.150–0.982)	0.046	
No	38	0.574 (0.143–2.297)	0.432	
LVEF≤35%				0.951
Yes	25	54.148 (0.001-5137,804)	0.495	
No	174	0.368 (0.162–0.836)	0.017	
Sex				0.143
male	122	0.719 (0.284–1.822)	0.487	
female	77	0.190 (0.042–0.870)	0.032	

Abbreviations: HR, hazard ratio; CI, confidence interval; LVEF: left ventricular eject fraction.

## Discussion

To our knowledge, this was the first study to evaluate levosimendan administration in patients undergoing TAVR using real-world data. Levosimendan administration had no effect on perioperative NT-proBNP and cTnI levels and 2-year all-cause mortality, However, it reduced in-hospital major adverse cardiac events (MACE) and 2-year stroke or HF-related hospitalization.

AS is the third-most frequent cardiovascular disease after CAD and hypertension (Lindman et al., 2016). Once the pathophysiologic cascade is initiated, the annual reduction in the valve area is approximately 0.1 cm. The LV afterload increases, resulting in sarcomere replication and subsequent concentric hypertrophy to maintain cardiac output (Steiner et al., 2017). LV hypertrophy reduces the compliance of the LV and impairs diastolic function. Eventually persistently elevated afterload and wall stress with LV hypertrophy cause LV wall fibrosis, oxygen supply-demand mismatch, and myocardial ischemia, leading to LV systolic failure. This mechanism is believed to be a major driver of the transition to symptoms, HF, and MACE (Boskovski and Gleason, 2021). However, the LVEF remains preserved in severe AS patients. In this study, the patients’ mean LVEF was 56.23%. Subclinical impairment of LV structure and function exist and correlate with symptom severity and outcome in patients whose LVEF is preserved. Substantial LV recovery may occur after AV replacement (Dahl et al., 2019). The potential cardiac damage indicates that proper inotrope administration is reasonable in AS patients. Landmark HF trials typically excluded hemodynamic significant AS. The role of drug therapy in the AS population is largely unknown. Since inotropic agents are contraindicated in patients with obstruction of LVOT, we started levosimendan infusion immediately after prosthesis implantation while LV afterload was reduced.

Since the first TAVR was performed in 2002, the technique has quickly developed. Unlike surgical AV replacement (SAVR), which requires cardiopulmonary bypass and cardioplegic arrest, TAVR can be performed percutaneously, leading to minor trauma and less damage to the heart. Levosimendan administration may be more effective in TAVR patients with less cardiac damage during operation.

Levosimendan can improve myocardial contractility without increasing oxygen demand. It can improve cardiac function in patients undergoing SAVR (Järvelä et al., 2008) or other cardiac surgery (De Hert et al., 2007; Leppikangas et al., 2011). Levosimendan has been evaluated in several studies to prevent and treat low cardiac output syndrome (LCOS) in patients who underwent cardiac surgery with the use of cardiopulmonary bypass. It is worth noting that all studies focused on levosimendan administration during operation have been based on conventional surgery (Papp et al., 2020). These findings may not be suitable to expand to minimally invasive TAVR.

Levosimendan administration in patients undergoing cardiac surgery remains controversial and different results have been obtained, in part due to differences in the following conditions (Haikala et al., 1995; Chen et al., 2021) systolic function, patients with or without pre-operative systolic dysfunction; drug administration, preoperative versus intraoperative versus postoperative; indication: preventing or treating LCOS; levosimendan doses: ranging from 0.025 to 0.2 μg/kg body weight/min with or without an initial bolus for the first 1 h; control group: administered placebo or other kinds of inotrope; and surgical method: coronary artery bypass graft or valve replacement. In this study, we did not exclude patients with preserved EF because only 25 patients had an LVEF of less than 35%. We started levosimendan infusion immediately after prosthesis implantation because it is contraindicated in patients with obstruction of LVOT and a previous study showed that early administration is associated with more benefits (Tasouli et al., 2007; Balzer et al., 2014). We chose 0.1 μg/kg bodyweight/min because loading doses and high-dose infusion are associated with hypotension and a less marked benefit (Landoni et al., 2012).

The major studies focused on levosimendan administration in patients undergoing cardiac surgery are three large, multicenter, randomized, placebo-controlled, trials published in 2017, namely the LEVO-CTS, CHEETAH, and LICORN trials. However, they failed to show that levosimendan improved survival rate or exerted renoprotective effects (Cholley et al., 2017; Landoni et al., 2017; Mehta et al., 2017). However, several post hoc analyses showed these positive results in certain patients (Zangrillo et al., 2018; van Diepen et al., 2020). These findings indicate that more studies are needed to identify patients who can benefit from levosimendan. Additionally, the safety of levosimendan administration was proven in these randomized controlled trials. The rates of hypotension, arial fibrillation, ventricular tachycardia or fibrillation, resuscitated cardiac arrest, and stroke did not significantly differ between the levosimendan group and the placebo group, consistent with our findings.

A previous study reported that levosimendan increases the risk of postoperative bleeding after cardiac valve surgery (Lahtinen et al., 2014). However, a recent study showed that levosimendan administration is not associated with an increased risk of bleeding and blood transfusion requirement in patients undergoing cardiac surgery (Wang et al., 2021). In this study, No major bleeding event was found, and minor bleeding events were unable to be evaluated because the medical record was insufficient for such events.

Kyrzopoulos et al. (2005) reported that levosimendan reduces plasma NT-proBNP in patients with severe HF. In our study, levosimendan showed no impact on NT-proBNP. The difference may result from a difference in HF severity between studies. Studies have also shown that an increase in cTnI after surgery is significantly lower in the LS + group. We also failed to find that levosimendan affects serum cTnI levels (De Hert et al., 2007; Tritapepe et al., 2009).

Levosimendan has been to shorten the length of ICU stay (Tritapepe et al., 2009) and hospital stay (Shah et al., 2014); reduce in-hospital adverse events (Lahtinen et al., 2011); and protect renal (Bragadottir et al., 2013), hepatic (Alvarez et al., 2013), and neural (Zangrillo et al., 2018) functions. However, these benefits remain controversial (Erb et al., 2014; Jawitz et al., 2021), as most of these findings are from small, single-center studies. In our study, we found no differences between the LS + group and LS- group regarding the length of ICU and hospital stays. In-hospital MACE was lower in the LS + group, but the number of events was too small to perform a multivariable analysis to exclude confounding factors.

A long-term survival benefit was not found in our study, consistent with previous reports (Lahtinen et al., 2011; Grieshaber et al., 2016). To the best of our knowledge, this was the first study to show that levosimendan can reduce long-term stroke or HF-related hospitalization in patients undergoing TAVR. An increasing number of cardiologists believe that crude mortality may not be the most important parameter to judge outcomes in studies on inotropic agents, because complex pathophysiology is unlikely to be moderated by a single intervention in such conditions. Isolated all-cause mortality should be addressed as a safety signal, not a primary marker of effectiveness. Our results may be interpreted as levosimendan is a safe and effective treatment in TAVR patients that reduces long-term stroke or HF-related hospitalization but does not increase all-cause mortality. Furthermore, levosimendan administration is considered a cost-effective strategy for reducing the incidence of postoperative low cardiac output and shortening ICU stay (Jiménez-Rivera et al., 2020). The economic evaluation of levosimendan administration in TAVR needs further study.

Our study had several limitations. First, this was a single-center retrospective study with loose criteria for the administration of levosimendan. However, after PSM, patients’ baseline was comparable between groups. Second, the sample size was small. Third, since Swan-Ganz catheterization is not necessary for patients undergoing TAVR, hemodynamic and organ function parameters were lacking in this study. Finally, since TAVR devices are relatively expensive (23,000 to 46,000 USD for one valve system versus 4723 USD for per capita disposal income in China, 2019) and not covered by national medical insurance, the financial situation and medical resources of patients who received TAVR were better than the general population, which caused selection bias.

## Conclusion

In this study, prophylactic levosimendan administration immediately after prothesis implantation in patients undergoing TAVR was found to reduce in-hospital MACE rates and stroke or HF-related hospitalization but did not result in lower all-cause mortality. More studies are needed to evaluate levosimendan administration in TAVR.

## Data Availability

The raw data supporting the conclusions of this article will be made available by the authors, without undue reservation.

## Abbreviations

AS, Aortic stenosis; TAVR, Transcatheter aortic valve replacement; SAVR, surgical aortic valve replacement; LVOT, left ventricular outflow tract; LV, left ventricle; AVR, Aortic valve replacement; LVEF, left ventricular ejection fraction; NT-proBNP, N-terminal pro-B-type natriuretic peptide; cTnI, cardiac troponin I; LDL-C, low density lipoprotein cholesterol; COPD, chronic obstructive pulmonary disease; CAD, coronary artery disease; LCOS, low cardiac output syndrome.
