# Supplementary material for: The effects of levosimendan in patients undergoing transcatheter aortic valve replacement- a retrospective analysis
Source: Front Pharmacol. 2022 Nov 3;13:969088. doi: 10.3389/fphar.2022.969088 (PMC9669067; doi:10.3389/fphar.2022.969088)
Supplement: Supplementary file 1 [file DataSheet1.docx]

Supplementary Material

**Supplementary Table1.** Baseline characteristics for unmatched study population

| parameter | LS- | LS+ |  |
| --- | --- | --- | --- |
|  | N=128 | N=157 | P value |
| Age, mean (SD), years | 75.84（6.89） | 77.11（6.19） | 0.102 |
| Male sex, n (%) | 79（61.7） | 94（59.9） | 0.751 |
| BMI, mean (SD), Kg/m^2^ | 23.59（3.34） | 23.09（3.83） | 0.252 |
| STS risk score, median [IQR] | 3.8[3.34] | 5.02[6.14] | **<0.001** |
| Heart failure, n (%) | 109（85.2） | 127（80.9） | 0.343 |
| NYHA class, n (%) |  |  |  |
| I, n (%) | 0（0） | 2（1.3） | 0.450 |
| II, n (%) | 11（8.6） | 18（11.5） |  |
| III, n (%) | 80（62.5） | 80（51） |  |
| IV, n (%) | 18（14.1） | 27（17.2） |  |
| Peak AV velocity, mean (SD), cm/s | 4.65(0.86) | 4.49(0.82) | 0.130 |
| LVEF, mean (SD), % | 57.16（14.456） | 55.36（13.577） | 0.283 |
| cTnI, median [IQR], ng/ml | 0.026[0.049] | 0.028[0.040] | 0.818 |
| NT-proBNP, median [IQR], pg/ml | 1678[2940] | 2063[5399] | **0.097** |
| diabetes, n (%) | 34（26.6） | 48（30.6） | 0.457 |
| CKD, n (%) | 8（6.3） | 12（7.6） | 0.647 |
| COPD, n (%) | 22（17.2） | 18（11.5） | 0.167 |
| hypertension, n (%) | 74（57.8） | 97（61.8） | 0.496 |
| dyslipidemia, n (%) | 67（47.7） | 99（63.1） | **0.068** |
| Previous CAD, n (%) | 55（43） | 56（35.7） | 0.209 |
| Previous AF, n (%) | 23（18） | 21（13.4） | 0.286 |
| statin, n (%) | 78（60.9） | 111（70.7） | **0.083** |
| aspirin, n (%) | 83（64.8） | 76（48.4） | **0.005** |
| β-block, n (%) | 77（60.2） | 98（62.4） | 0.696 |
| ACEI/ARB, n (%) | 21（16.4） | 28（17.8） | 0.751 |
| CCB, n (%) | 14（10.9） | 20（12.7） | 0.641 |
| digoxin, n (%) | 2（1.6） | 2（1.3） | 0.837 |

Abbreviations: BMI, body mass index; STS, Society of Thoracic Surgeons; NYHA, New York Heart Association; AV, aortic valve; LVEF, left ventricular ejection fraction; cTnI, cardiac troponin I; NT-proBNP, N-terminal pro-B-type natriuretic peptide; CKD, chronic kidney disease; COPD, chronic obstructive pulmonary disease; CAD, coronary heart disease; AF, atrial fibrillation; ACEI/ARB, angiotensin converting enzyme inhibitor/ angiotensin receptor blocker; CCB, calcium channel blocker.

**Supplementary Table2.**Univariablee cox proportional hazard analyses for 2-years all-cause mortality

| All-cause mortality | Hazard ratio (95% CI) | P value |
| --- | --- | --- |
| Age, per 1 year increase | 1.058(0.966-1.158) | 0.226 |
| Male, yeas/no | 0.733(0.246-2.182) | 0.577 |
| BMI, per 1kg/m^2^ increase | 0.972(0.825-1.145) | 0.733 |
| STS risk score, (log) | 0.990(0.812-1.207) | 0.922 |
| Peak AV velocity,per 1m/s increase | 1.567(0.741-3.313) | 0.240 |
| LVEF, per 1% increase | 1.039(0.986-1.095) | 0.152 |
| Heart failure, yes/no | 0.790(0.217-2.870) | 0.720 |
| NT-proBNP (log) | 0.828(0.335-2.047) | 0.683 |
| cTnI, (log) | 0.910(0.303-2.735) | 0.867 |
| COPD, yes/no | 4.019(1.315-12.289) | **0.015** |
| Diabetes, yes/no | 1.922(0.646-5.720) | 0.240 |
| Stroke, yes/no | 1.915(0.527-6.958) | 0.324 |
| hypertension, yes/no | 2.404(0.662-8.734) | 0.183 |
| dyslipidemia, yes/no | 1.841(0.567-5.978) | 0.310 |
| Previous CKD, yes/no | 0.955(0.124-7.343) | 0.965 |
| Previous CAD, yes/no | 1.222(0.411-3.636) | 0.718 |
| Previous AF, yes/no | 0.370(0.048-2.849) | 0.340 |
| Levosimendan, yes/no | 0.603(0.197-1.844) | 0.375 |

Abbreviations: BMI, body mass index; STS, Society of Thoracic Surgeons; AV, aortic valve; LVEF, left ventricular ejection fraction; cTnI, cardiac troponin I; NT-proBNP, N-terminal pro-B-type natriuretic peptide; CKD, chronic kidney disease; COPD, chronic obstructive pulmonary disease; CAD, coronary heart disease; AF, atrial fibrillation.

**Supplementary Table3.** Univariable cox proportional hazard analyses for 2-years Stroke or heart failure related-hospitalization.

| Stroke or heart failure related-hospitalization | Hazard ratio (95% CI) | P value |
| --- | --- | --- |
| Age, per 1 year increase | 1.044(0.976-1.117) | 0.214 |
| Male, yeas/no | 0.912(0.390-2.133) | 0.831 |
| BMI, per 1kg/m^2^ increase | 1.068(0.943-1.209) | 0.299 |
| STS risk score, (log) | 0.962(0.822-1.125) | 0.625 |
| Peak AV velocity,per 1m/s increase | 1.026(0.599-1.757) | 0.926 |
| LVEF, per 1% increase | 1.005(0.973-1.037) | 0.778 |
| Heart failure, yes/no | 0.495(0.202-1.214) | 0.125 |
| NT-proBNP (log) | 0.960(0.479-1.925) | 0.909 |
| cTnI, (log) | 0.969(0.421-2.228) | 0.940 |
| COPD, yes/no | 0.956(0.285-3.260) | 0.954 |
| Diabetes, yes/no | 2.880(1.244-6.668) | **0.014** |
| Stroke, yes/no | 2.455(0.960-6.275) | **0.061** |
| hypertension, yes/no | 1.263(0.530-3.012) | 0.598 |
| dyslipidemia, yes/no | 1.158(0.495-2.710) | 0.735 |
| Previous CKD, yes/no | 1.132(0.265-4.844) | 0.867 |
| Previous CAD, yes/no | 2.586(1.085-6.166) | **0.032** |
| Previous AF, yes/no | 2.799(1.174-6.676) | **0.020** |
| Levosimendan, yes/no | 0.346(0.135-0.884) | **0.027** |

Abbreviations: BMI, body mass index; STS, Society of Thoracic Surgeons; AV, aortic valve; LVEF, left ventricular ejection fraction; cTnI, cardiac troponin I; NT-proBNP, N-terminal pro-B-type natriuretic peptide; CKD, chronic kidney disease; COPD, chronic obstructive pulmonary disease; CAD, coronary heart disease; AF, atrial fibrillation.

**Supplementary Table4.** Univariable cox proportional hazard analyses for combined endpoint

| Combined endpoint | Hazard ratio (95% CI) | P value |
| --- | --- | --- |
| Age, per 1 year increase | 1.033(0.976-1.094) | 0.261 |
| Male, yeas/no | 0.948(0.457-1.969) | 0.887 |
| BMI, per 1kg/m^2^ increase | 1.015(0.912-1.129) | 0.787 |
| STS risk score, (log) | 0.956(0.834-1.095) | 0.512 |
| Peak AV velocity,per 1m/s increase | 1.099(0.691-1.748) | 0.689 |
| LVEF, per 1% increase | 1.007(0.976-1.036) | 0.616 |
| Heart failure, yes/no | 0.646(0.287-1.450) | 0.289 |
| NT-proBNP (log) | 0.935(0.517-1.690) | 0.823 |
| COPD, yes/no | 1.551(0.634-3.794) | 0.336 |
| Diabetes, yes/no | 2.392(1.169-4.895) | **0.017** |
| Stroke, yes/no | 2.462(1.096-5.534) | **0.029** |
| hypertension, yes/no | 1.450(0.678-3.097) | 0.338 |
| dyslipidemia, yes/no | 1.204(0.580-2.500) | 0.618 |
| Previous CKD, yes/no | 0.796(0.190-3.342) | 0.756 |
| Previous CAD, yes/no | 1.918(0.931-3.950) | **0.077** |
| Previous AF, yes/no | 1.748(0.778-3.927) | 0.176 |
| Levosimendan, yes/no | 0.459(0.215-0.980) | **0.044** |

Abbreviations: BMI, body mass index; STS, Society of Thoracic Surgeons; AV, aortic valve; LVEF, left ventricular ejection fraction; cTnI, cardiac troponin I; NT-proBNP, N-terminal pro-B-type natriuretic peptide; CKD, chronic kidney disease; COPD, chronic obstructive pulmonary disease; CAD, coronary heart disease; AF, atrial fibrillation.
